# Targeting cyclin-dependent kinases in sarcoma treatment: Current perspectives and future directions

**DOI:** 10.3389/fonc.2023.1095219

**Published:** 2023-01-19

**Authors:** Alessandra Merlini, Valeria Pavese, Giulia Manessi, Martina Rabino, Francesco Tolomeo, Sandra Aliberti, Lorenzo D’Ambrosio, Giovanni Grignani

**Affiliations:** ^1^ Candiolo Cancer Institute, IRCCS-FPO, Turin, Italy; ^2^ Department of Oncology, University of Turin, Turin, Italy; ^3^ Medical Oncology, Azienda Ospedaliera Universitaria San Luigi Gonzaga, Turin, Italy

**Keywords:** sarcoma, cyclin dependent kinases (CDK), cdk inhibitors, target therapy, cell cycle

## Abstract

Effective treatment of advanced/metastatic bone and soft tissue sarcomas still represents an unmet medical need. Recent advances in targeted therapies have highlighted the potential of cyclin-dependent kinases (CDK) inhibitors in several cancer types, including sarcomas. CDKs are master regulators of the cell cycle; their dysregulation is listed among the “hallmarks of cancer” and sarcomas are no exception to the rule. In this review, we report both the molecular basis, and the potential therapeutic implications for the use of CDK inhibitors in sarcoma treatment. What is more, we describe and discuss the possibility and biological rationale for combination therapies with conventional treatments, target therapy and immunotherapy, highlighting potential avenues for future research to integrate CDK inhibition in sarcoma treatment.

## 1 Introduction

Sarcomas are a heterogeneous group of rare, mesenchymal malignancies that add up to 1% of all adult cancers and 20% of pediatric cancers (1). The sarcoma family encompasses more than 100 histological subtypes, comprising bone sarcomas and soft tissue sarcomas (BSTS) (2). Standard treatment entails radical surgical resection with (neo)adjuvant radiation therapy and/or chemotherapy in high-risk patients for localized disease, and systemic chemotherapeutic treatment in advanced stages (3). However, prognosis in advanced/metastatic stages remains dismal for the vast majority of sarcoma patients (3, 4). Hence, finding novel, effective treatment strategies for advanced BSTS represents an unmet medical need. Indeed, differently from epithelial cancers, mesenchymal tumors have seldom benefitted from the advent of innovative therapeutic strategies, from targeted therapy to immunotherapy (5, 6). Both the rarity of sarcomas, and the variety of their molecular determinants (7, 8), have represented major challenges for the development of effective, innovative therapeutic strategies in the field in past years. One of the first actionable molecular alterations discovered in sarcomas has been the amplification of the chromosomal region encoding the murine double minute 2 (MDM2) and cyclin dependent kinase 4 (CDK4) genes in a subset of liposarcomas (well-differentiated and dedifferentiated liposarcomas; WDLPS and DDLPS) (9, 10).

However, until recent years, the possibility to safely target master regulators of the cell cycle as MDM2 and CDK4 appeared difficult to translate in the clinical setting, for their potential off-tumor side effects in healthy tissues (11, 12). Targeting CDK4 seemed particularly attractive for WDLPS and DDLPS, in which it has a specific clinical and biological significance, with respect to MDM2 amplified-only liposarcomas. Indeed, CDK4-amplified WDLPS and DDLPS have been associated with worse prognosis with respect to those lacking CDK4 amplification (13). However, the CDK family is involved not only in WDLPS and DDLPS pathobiology, but in many different sarcoma types across BSTS (14). The comprehensive genomic analysis *via* The Cancer Genome Atlas (TCGA) has shown that approximately one quarter of all sarcomas harbor genetic alterations in the Cyclin-Dependent Kinase Inhibitor 2A (CDKN2A) - Cyclin D (CCND) - CDK4 - retinoblastoma (RB) axis (15, 16), providing strong rationale for targeting this crucial pathway in sarcomas. Hence, better understanding of the role of CDKs in cell biology and cancer, might provide novel avenues of treatment for advanced BSTS (14, 17).

## 2 CDKs in physiology and cancer

The cell cycle is divided into four distinct phases: a first growth phase (G1), a DNA replication or synthesis phase (S), a second growth phase (G2) and the mitotic phase (M). Cyclin-dependent kinases (CDKs) are members of the serine/threonine protein kinase family; as master regulators of cell cycle control, transcription, and RNA splicing, they are essential for tumor cell proliferation and growth. CDKs do not possess autonomous enzymatic activity and need to be bound to a cyclin subunit to function properly, hence their designation as cyclin-dependent kinases (18). Moreover, a few CDK family members play an important role in RNA transcription and pre-messenger RNA (mRNA) splicing.

The activity of CDKs is respectively up and down regulated by their cyclin partners and cyclin-dependent kinase inhibitors (CKIs). CDKs can phosphorylate the tumor suppressor protein retinoblastoma (Rb). This activity blocks the growth-inhibitory function of Rb: indeed, phospho-Rb (pRb) releases its grip, previously blocking the transactivation domain of the E2F transcription factors, allowing the transcription of genes which are crucial for cell cycle progression to the S-phase (19). In detail, cyclin D-CDK4/6 kinase complexes phosphorylate multiple Rb tumor suppressor protein residues (or its homologs, p107 and p130). As abovementioned, in its hypo-phosphorylated state, Rb actively suppresses G1-S progression by sequestering E2F transcription factors, which transcribe genes needed for DNA replication (20).

The human genome encodes 20 CDKs, divided into two subfamilies: cell cycle-associated CDKs (CDK1−7 and CDK14−18) and transcription-associated CDKs (CDK7−13, 19, and 20). Different CDKs interact with different cyclins to regulate numerous stages of the cell cycle in various cells or to perform other functions. CDK1 is the ancestor of all mitotic kinases; CDK2, CDK4, and CDK6 regulate progression through cell cycle phases. CDK7, instead, is peculiar in that it has been implicated in both transcription processes and cell cycle control (21). CDK8 and CDK9 control the RNA polymerase II (RNA Pol II)-dependent initiation and elongation of transcription (22). Other CDKs (5, 10, 11, 14–18, and 20) do not fit into either canonical roles, exhibiting different functions, often in a tissue-specific fashion. For example, CDK11 has multiple functions in mediating apoptosis, transcription, mitosis, hormone receptor signaling, and autophagy (23, 24). Likewise, CDK5 promotes neuron outgrowth and synaptogenesis in the nervous system, while in pancreatic β cells it reduces insulin secretion (25). As CDKs master fundamental processes required for cell survival and propagation, their hyperactivation (typically through mutation, gene amplification, or altered expression of their regulators) is frequently reported in cancer.

Until a few years ago, CKIs were also classified in two families of cell cycle inhibitors: the CDK family interacting with the CIP/KIP protein and the kinase inhibitor (INK) family. CIP/KIP family members are specific for CDK-cyclin complexes, such as CDK2-cyclin E, A and/or CDK1-cyclin B1, A and/or CDK2,4,6-cyclin D1, D2, D3. Members of the INK family bind CDK4,6 to inhibit formation of CDK4,6-cyclin D1, D2, D3 complexes (26, 27).

## 3 Partners in life, partners in crime: Key players in cell cycle function and dysregulation beyond CDKs

More recently, additional important regulatory proteins and mechanisms involved in cell cycle control have been discovered, such as members of CDK regulatory subunit (CKS) protein family and new cell cycle regulators. A recent addition to the family is the double homeobox 4 (DUX4) protein, which is of specific interest for sarcoma pathobiology. DUX4 is a transcription factor physiologically expressed during early embryonic development, and it is silenced by epigenetic pathways in most adult somatic cells. Studies revealed that DUX4 binds to CDK1, preventing the formation of CDK1-cyclin B1 complex, thus limiting its kinase activity (28). Aberrant expression of DUX4 in skeletal muscle leads to facioscapulohumeral dystrophy (26, 29). DUX4 rearrangements have been identified in specific types of pediatric B cell acute lymphoblastic leukemia (30, 31), in small round cell bone and soft tissue sarcomas – the so-called CIC-DUX4 rearranged family of sarcomas (32, 33), and rhabdomyosarcoma (34).

The dysregulation of CDK activity through activation of pathways enhancing CDK activity, or through the oncogene-induced inactivation of apoptosis, is a common occurrence in various cancers (35). Identifying and characterizing which cancer types require selected CDK activities for proliferation and survival, might enable to understand which subtypes could benefit more from specific CDK inhibitors (CDKi). However, weighing the importance of each CDK activity to cancer initiation, proliferation and progression is no trivial task, given the individual, multiple roles of each CDK and cyclin beyond cell cycle control (36).

In cancer, CDKs affect multiple targets and phosphorylate relevant transcription factors involved in tumorigenesis. What is more, their pathway can be altered at different stages in various cancer subtypes; even within the same cancer type (and, most importantly, within the same patient), multiple CDK pathway alterations can co-exist and, in some cases, provide escape/resistance to CDK inhibition. Moreover, resistance almost invariably ensues with targeted treatments in cancer, due to both intratumor heterogeneity and tumor evolutionary dynamics, and CDKi treatment is no exception to the rule. The emergence of somatic RB mutations has been identified in the clinic as a relevant resistance mechanism in breast cancer patients treated with CDKi (37); RB mutation/deletion is a frequent event in sarcomas, with deep deletions detected in a significant proportion of STS in the TCGA sarcoma cohort (16).

Another commonly deleted key tumor suppressor gene is Cyclin Dependent Kinase Inhibitor 2A (CDKN2A). CDKN2A encodes two important cell cycle regulatory proteins, p16 (encoded by the INK4A gene) and, in an alternative reading frame, p14 (encoded by the Alternative Reading Frame – ARF - gene). CDKN2A deletions and inactivating mutations seem to have a negative prognostic role across different tumor types, including sarcomas (38–42). p16, a CDK inhibitor, inhibits Rb phosphorylation, while p14 inhibits MDM2, resulting in a positive regulation of p53. p16 expression increases gradually to a sustained, significantly high level in the later stages of cellular senescence.

Indeed, in murine cells, p19/p53 pathway inactivation is generally sufficient to escape senescence, while in human cells disruption of at least both the p53/p21 and the p16/pRb pathways is usually needed. Homozygous deletion of CDKN2A/ARF thus results in inactivation of two major tumor suppressing pathways, mainly acting through Rb and p53 (43).

**Figure 1:**
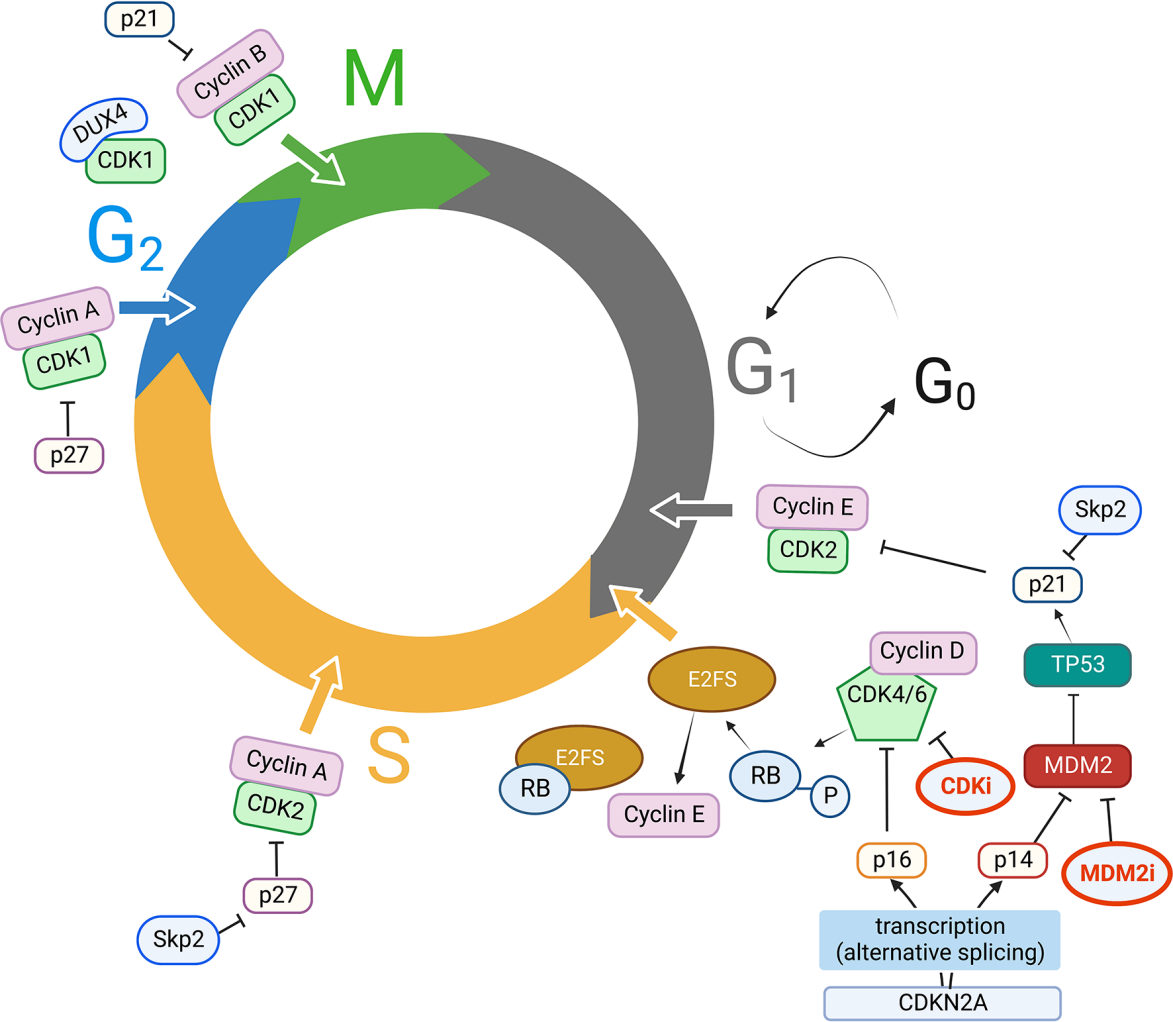
Key players in cell cycle dysregulation in STS. CDK, Cyclins, CKI, and other key molecular players in CDK activity/inhibition. Clockwise, starting from G1 to S phase progression: CDKN2A is transcribed by alternative splicing either into p16 or p14, which respectively inhibit CDK4/6/Cyclin D complexes and MDM2 activity. MDM2, an ubiquitin ligase, ubiquitinates p53 targeting it to the proteasome; p53 has p21 as a direct transcriptional target, and p21 in turn inhibits CDK2/Cyclin E complexes. Cyclin E expression is regulated by E2F transcription factor, which in turn is released from Rb protein grip (usually blocking its transactivation domain) when CDK4/6/Cyclin D complexes phosphorylate Rb, facilitating G1 to S phase progression. The ubiquitin ligase Skp2 targets p21 and p27 for proteasomal degradation, thus promoting CDK2/Cyclin E, CDK2/Cyclin A activity in S phase progression, CDK1/Cyclin A activity for G2 to M transition, which is also fostered by releasing p21 inhibitory activity on CDK1/Cyclin B; DUX4 can also bind CDK1, thus preventing CDK1/Cyclin B interaction. MDM2 activity can also be inhibited with MDM2 inhibitors (MDM2i), while CDK inhibitors (CDKi) currently in use in clinical practice are mainly CDK4/6 inhibitors. MDM2i and CDKi are highlighted in bold (red) in **Figure 1**. *Created with BioRender.com*.

Hence, a plethora of alterations beyond CDK4/6 genes emerges as highly relevant for sarcoma pathobiology, providing several potential actionable targets at various steps of the CDKN2A-CCND-CDK4-RB axis. Understanding which sarcoma subtypes are most affected by specific alterations in this axis, has provided the rational basis to select those sarcomas which could benefit more from CDK inhibition (14) (**Figure 1**).

## 4 Actionable targets in CDK signaling across different sarcoma subtypes

Despite the diversity in histotypes, age at presentation, risk of recurrence and prognosis, the most frequently altered genes in sarcomas precisely include genes involved in cell cycle regulation, namely TP53, CDKN2A, RB (44–46). Surprisingly, the only gene whose alterations were associated with worse overall survival across all types of STS was CDKN2A (39). These results confirm the biological importance of the p16INK4a-CDK4/6-pRb pathway and/or ARF signaling pathways in sarcoma (39). Indeed, pinpointing histotype-specific alterations might help to dissect the most appropriate therapeutic challenges and opportunities for each sarcoma subtype.

### 4.1 Undifferentiated pleomorphic sarcoma

UPS accounts for 15–20% of all STS. Typically, it occurs in the limbs and trunk of adults >40 years of age (47). The development of most UPS is sporadic, but approximately 3% of UPS develop in areas of the body that received radiation therapy to treat an unrelated disease after a median latency of 10 years, and are consequently classified as secondary (or, more appropriately, radiation-induced) UPS (48). The standard of care for patients with localized UPS is surgical resection with (neo)adjuvant chemo/radiotherapy in selected cases; for patients with unresectable or metastatic disease, systemic chemotherapy and/or radiotherapy may be considered with low to moderate response rates in patients with UPS. Remarkably, UPS are also among the most represented sarcoma histotypes with CDKN2A loss (39). About 30% of UPS show MDM2 and CDK4 up-regulation; MDM2 ubiquitinates the tumor suppressor p53 and promotes its proteasomal degradation, while MDM2 overexpression leads to downregulation of the CKI p21. P21 is a transcriptional target of p53, and its downregulation causes hyperactivation of CDKs (49). Up to 78% of UPS tumors carry RB gene deletions, due to losses of different regions within the long arm of chromosome 13 (8). TP53 is also very frequently deleted in UPS, and together with RB and ATRX, is among the few genes recurrently showing pathogenic missense mutations in UPS (16). Intriguingly, S phase kinase-associated protein 2 (Skp2) is required for survival of RB-and TP53-deficient UPS cells, in which it drives cell proliferation by degrading p21 and p27. Hence, the loss of both RB and TP53 renders UPS dependent on Skp2, which could provide the basis for innovative therapeutic strategies in this setting (50). However, there are no experimental studies ongoing or published so far, about the potential of CKIs in UPS patients.

### 4.2 Liposarcoma

Liposarcomas (LPS) account for a significant proportion (~13–20%) of adult STS (13-20%). LPS are subcategorized into three main groups, including WDLPS/DDLPS, characterized by a typical MDM2 and high-mobility group AT-hook 2 (HMGA2) gene amplification and an inconsistent CDK4 gene amplification (the 12q amplicon can span chromosomal regions from 12q12 up to 12q21); myxoid/round cell liposarcoma (M/RCLPS), carrying a typical t (12, 16)(q13;p111) translocation, and pleomorphic liposarcoma (PLPS), frequently showing TP53 and/or RB gene losses. Roughly 60% of LPS cases are WD/DDLPS, while PLPS is the rarest subtype (~5%). In WDLPS/DDLPS, the CDK4 gene (12q14.1) is within a distinct, inconsistent amplicon that is not present in about 10% of WDLPS/DDLPS (51), and its presence has been associated with a worse prognostic outcome (13). Moreover, patients carrying both gene amplifications (MDM2 and CDK4) have a much higher risk of local recurrence after surgery. The WDLPS/DDLPS genetic signature shows a complex pattern of expression for Cyclin D1, P16INK4a, P14ARF, and RB which is not dependent on CDK4 status. Finally, alterations in CDKN2A/CDKN2B/CDK4/CCND2 axis have been detected in almost all CDK4 amplification-negative WDLPS/DDLPS in a cohort of 104 WDLPS/DDLPS patients (52).

### 4.3 Malignant peripheral nerve sheath tumors

Malignant peripheral nerve sheath tumors (MPNSTs) add up to 3-10% of all STS diagnoses. They can arise sporadically or in patients affected by neurofibromatosis type I (NF1). MPNSTs are very aggressive and the first cause of oncological death in patients affected by NF1. In those tumors, CDK2 and CDK 4/6 are overexpressed because of the loss of p16 and p27. This causes constant pRb phosphorylation, fostering cell cycle progression (53). Remarkably, up to 80% of MPNST show CDKN2A loss (54). This leads to the upregulation of CDK4/6 and sequentially the initiation of the S phase and promotion of mitosis. Hence, CDK4/6 inhibitors (CKIs) hold promise as a potential innovative treatment for advanced MPNST (55).

### 4.4 Synovial sarcoma

Synovial sarcoma typically arises in young adults, and is characterized by a typical translocation between chromosome X and chromosome 18 t(X,18;p11,q11), which generates a fusion between SS18 and SSX1/2 or SSX4, disrupting epigenetic regulation within the cancer cell (56, 57). CDKN2A deletion is a highly frequent event in synovial sarcomas (58); moreover, the translocation facilitates repression of CDKN2A activity (59) and increases the expression of CDK4 as well as multiple cyclins (D1, B1, A2, I, and F) (60).

### 4.5 Other soft tissue sarcomas

*Leiomyosarcoma (LMS)* accounts for 10-20% of all STS, and can arise at any body site. LMS is characterized by spindle-shaped cells resembling smooth muscle cells and are grouped among the so-called “complex karyotype” STS, as they are not driven by a single translocation or genetic alteration, but are characterized by multiple, various genetic abnormalities. Common genetic alterations include PTEN deletion and/or mutation, TP53 mutations and, importantly, RB loss (16, 61–63). One striking, recent finding is the high frequency of biallelic inactivation of the above mentioned by various mechanisms, in the vast majority of LMS samples analyzed in the study by *Chudasama P.* and colleagues (61). Rb inactivation casts some doubt on the clinical utility of CDK inhibition, as Rb inactivation affects the CDK pathway downstream of CDKs, presenting a potential mechanism of both primary and secondary resistance to CDKi in LMS, similarly to Rb-mediated resistance mechanisms in CDK-treated breast cancer patients (37).

*Intimal sarcomas (INS)* are rare STS which can be particularly aggressive also because of their site of origin, most frequently affecting the wall of large vessels or the heart (64–66). INS are characterized by the peculiar presence of large gains/amplifications in the 12q12-15 chromosomal region, encompassing MDM2 and/or CDK4. CDKN2A deletions are also very frequent in INS (65). Taken together, these recent molecular findings might provide the rationale for trials with CDKi in this set of STS patients burdened by very poor prognosis (67).

*Rhabdomyosarcoma (RMS)* is the most common STS in children and adolescents; alveolar rhabdomyosarcomas (ARMS) are characterized by either PAX3-FOXO1 or PAX7-FOXO1 fusion genes; ARMS with the former fusion most often carry additional 12q13-q14 amplifications, therefore including the CDK4 gene, which has been correlated with poor survival outcomes (68). Disappointingly, in fusion-positive RMS, CDK4 amplification has not been linked to increased sensitivity to CDKi, but, rather counterintuitively, to the opposite condition (resistance to CDKi), at least in *in vitro* studies (69).

### 4.6 Bone sarcomas

*Osteosarcomas (OS)* represent the most common primary malignant tumors of bone. They can arise at any skeletal site, but they more frequently develop in the long bones of the extremities. OS has a bimodal age distribution (adolescents between 14-18 years and older adults, > 40 years old) and, even though it is sensitive to chemotherapy, prognosis in advanced stages remains dismal (2, 70). Intriguingly, TP53 is inactivated in >90% of OS, contributing to cell cycle dysregulation; RB1 is also among genes most frequently mutated in OS (>50%) (71). Indeed, individuals affected by Li-Fraumeni and hereditary retinoblastoma syndromes have an increased risk of developing OS (72). Other genes commonly altered in OS and involved in cell cycle regulation include CDK4, MDM2, PTEN, CDKN2A, CCND3, and CCNE1 (14, 49, 73). The clinical utility of CDKi in OS has not yet been tested in dedicated clinical trials, but advanced osteosarcoma patients with CDK4 overexpression could be included in the phase II PalboSarc trial with the CDKi palbociclib (NCT03242382) (14).

## 5 Targeting CDKs in cancer

The history and success of CDKi in cancer have now come a long way, with more than 25 years of preclinical and clinical development (74). The first generation of CDKi was constituted by pan-CDKi (e.g. flavopiridol, olomucine, roscovitine) (75, 76), which were designed to halt cell cycle and cell proliferation by inhibiting CDK enzymatic activity. This first generation of pan-CDKi had limited selectivity and was burdened by high toxicity in normal cells, preventing their clinical development. For these reasons, almost all first generation CDKi failed to meet their endpoints in early-stage clinical trials (77, 78). Second-generation CDKi (e.g. dinaciclib, CYC065) have been developed with greater selectivity and fewer side effects (79). Finally, third-generation, selective CDK4/6 inhibitors were the first CDKi which received FDA approval in March 2017, for the treatment of postmenopausal women with hormone receptor (HR)-positive metastatic breast cancer, in combination with an aromatase inhibitor as initial endocrine-based therapy.

Currently, the three FDA- and EMA-approved CDK4/6 inhibitors are palbociclib, ribociclib and abemaciclib. While palbociclib is equally active against CDK4 and CDK6, ribociclib and abemaciclib show higher efficacy in CDK4 inhibition (80). Indeed, all these approved compounds act by inhibiting Rb phosphorylation, thus blocking cell cycle progression from G1 to S phase. However, their action extends beyond simple enzymatic inhibition, with likely direct effects on cell metabolism, senescence, and possibly immune modulation (81, 82).

### 5.1 Anti-CDK targeted therapy in sarcomas: Ongoing clinical trials and future perspectives

Sarcomas have been included in clinical studies on CDKi since early phase I trials; however, only very few CDKi trials enrolled exclusively BSTS, including an early-phase trial of flavopiridol in association with doxorubicin (14, 75). Among these, two studies are of peculiar interest. In particular, the study “PD0332991 (Palbociclib) in Patients with Advanced or Metastatic Liposarcoma”, NCT01209598, demonstrated a favorable progression free survival (PFS) in a mixed WDLPS/DDLPS patient cohort, which included advanced/metastatic WDLPS/DDLPS patients who had received at least one line of systemic treatment (patients previously untreated for systemic disease were allowed to join the expansion cohort) (83). Another study, NCT02846987, still active although closed to enrollment, has investigated the role of abemaciclib monotherapy in advanced DDLPS, assuming that this novel, more potent CDK inhibitor might achieve better results in the sarcoma population. So far, the study has met its primary endpoint (12-week PFS ≥ 60%) and final results are awaited (84).

One highly attractive combination treatment opportunity in WDLPS/DDLPS is represented by the possibility to combine novel MDM2 inhibitors (85, 86) with CDKi. Preclinical studies demonstrated both evidence of synergism (87), and efficacy of MDM2 inhibitors in overcoming resistance to CDK4/6 inhibitors (88). However, the significant risk of unacceptable combined toxicities of MDM2 and CDK4 inhibitors - especially myelosuppression - casts some doubt over the clinical applicability of their combination.

Concerning other possible targeted treatment combinations, one interesting opportunity could be the association of CDKi with PI3K inhibitors. Indeed, PTEN downregulation and AKT increased phosphorylation were shown to be associated with increased CDK2/cyclin E2 expression in breast cancer cell lines resistant to CDKi, rendering PI3K inhibitors (capable of downregulating cyclin E2) an attractive partner to overcome resistance to CDKi (89).

Finally, studies on the association (combination/sequence) of CDKi with immunotherapy are currently ongoing in many cancer types, including sarcomas (e.g. study NCT04438824, listed in **Table 1**). Indeed, CDKi seem to have a relevant immune-priming effect (81). Preliminary data are not available yet for BSTS, but similar studies in breast cancer with combination of palbociclib, pembrolizumab and letrozole have yielded promising results (90).

**Table 1:** Ongoing clinical trials with CDKi in BSTS (source: www.clinicaltrials.gov, accessed on October 20^th^, 2022).

ClinicalTrials.gov identifier	Study Title	Status	Interventions	Study Type, Phase
NCT03242382	Trial of Palbociclib in Second Line of Advanced Sarcomas with CDK4 Overexpression.	Recruiting	• Drug: Palbociclib	Study Type: Interventional Phase: Phase II
NCT04040205	Abemaciclib for Bone and Soft Tissue Sarcoma with Cyclin- Dependent Kinase (CDK) Pathway Alteration	Recruiting	• Drug: Abemaciclib	Study Type: Interventional Phase: Phase II
NCT03604783	Phase 1, First-in-human Study of Oral TP-1287 in Patients with Advanced Solid Tumors	Recruiting	• Drug: TP-1287	Study Type: Interventional Phase: Phase I
NCT05159518	A Study of PRT2527 in Patients with Advanced Solid Tumors	Recruiting	• Drug: PRT2527	Study Type: Interventional Phase: Phase I
NCT04941274	Abemaciclib in Patients With HIV-associated and HIV-negative Kaposi Sarcoma	Recruiting	• Drug: Abemaciclib	Study Type: Interventional Phase: Phase I-II
NCT02644460	Abemaciclib in Children with DIPG or Recurrent/Refractory Solid Tumors	Recruiting	• Drug: Abemaciclib	Study Type: Interventional Phase: Phase I
NCT04557449	Study to Test the Safety and Tolerability of PF-07220060 in Participants with Advance Solid Tumors	Recruiting	• Drug: PF-07220060	Study Type: Interventional Phase: Phase I
NCT04438824	Palbociclib and INCMGA00012 in People with Advanced Liposarcoma	Recruiting	• Drug: INCMGA00012	Study Type: Interventional Phase: Phase II
NCT03784014	Molecular Profiling of Advanced Soft-tissue Sarcomas	Recruiting	• Drug: Nilotinib • Drug: Ceritinib • Drug: Capmatinib • Drug: Lapatinib • Drug: Trametinib • Combination Product: Trametinib and Dabrafenib • Combination Product: Olaparib and Durvalumab • Drug: Palbociclib • Drug: Glasdegib • Drug: TAS-120 • Other: Next Generation sequencing exome	Study Type: Interventional Phase: Phase III
NCT05252416	(VELA) Study of BLU-222 in Advanced Solid Tumors	Recruiting	• Drug: BLU-222 • Drug: Carboplatin • Drug: Ribociclib • Drug: Fulvestrant	Study Type: Interventional Phase: Phase I-II
NCT03709680	Study Of Palbociclib Combined with Chemotherapy In Pediatric Patients With Recurrent/Refractory Solid Tumors	Recruiting	• Drug: Palbociclib	Study Type: Interventional Phase: Phase II
NCT04238819	A Study of Abemacicli (LY2835219) in Combination with Other Anti-Cancer Treatments in Children and Young Adult Participants With Solid Tumors, Including Neuroblastoma	Recruiting	• Drug: Abemaciclib	Study Type: Interventional Phase: Phase I-II

In **Table 1**, a list of ongoing, actively recruiting clinical trials with CDKi (alone as monotherapy, or in combination) in BSTS is provided.

## 6 Conclusions

The presence of molecular alterations affecting the CDKN2A-CDK4-CCND1-RB axis is an important opportunity for innovative targeted treatments for patients with BSTS, typically burdened by dismal prognosis in advanced/metastatic stages. Knowledge of the fine-tuning of these pathways across different sarcoma subtypes is instrumental to develop rationally-based clinical trial proposals in this setting. Indeed, presence of multiple alterations in different steps of cell cycle regulation might provide primary/secondary resistance mechanisms to CDK inhibition; moreover, when present, CDK4 amplification is the main oncogenic driver of only a subset of CDK4-amplified sarcomas. Hence, thorough understanding of the molecular basis of cell cycle dysregulation in each specific histotype, will be crucial for the development of tailored treatment combinations with CDK inhibitors and other innovative targeted therapies or immunotherapeutic strategies.

## Author contributions

AM, LA, and GG contributed to conception and design of the review article. AM, VP, LA organized the literature data. AM, VP, LA and GG wrote the first draft of the manuscript. AM, VP, GM, MR, FT, SA, LA, GG wrote sections of the manuscript. All authors contributed to the article and approved the submitted version.
